# Receptor Interactions of Angiotensin II and Angiotensin Receptor Blockers—Relevance to COVID-19

**DOI:** 10.3390/biom11070979

**Published:** 2021-07-03

**Authors:** Graham J. Moore, Jose M. Pires, Konstantinos Kelaidonis, Laura Kate Gadanec, Anthony Zulli, Vasso Apostolopoulos, John M. Matsoukas

**Affiliations:** 1 Pepmetics Inc., 772 Murphy Place, Victoria, BC V8Y 3H4, Canada; 2 Department of Physiology and Pharmacology, Cumming School of Medicine, University of Calgary, Calgary, AB T2N 1N4, Canada; 3 Department of Physics, Federal University of Espirito, Santo, Vitoria 29075-910, Brazil; pires2004@hotmail.com; 4 NewDrug, Patras Science Park, 26500 Patras, Greece; k.kelaidonis@gmail.com; 5 Institute for Health and Sport, Victoria University, Melbourne, VIC 3030, Australia; laura.gadanec@live.vu.edu.au (L.K.G.); anthony.zulli@vu.edu.au (A.Z.); vasso.apostolopoulos@vu.edu.au (V.A.)

**Keywords:** ACE2, angiotensin II, AT1R, charge relay system, COVID19, EXP3174, SARS-CoV-2, sartans

## Abstract

Angiotensin II (Ang II) may contain a charge relay system (CRS) involving Tyr/His/carboxylate, which creates a tyrosinate anion for receptor activation. Energy calculations were carried out to determine the preferred geometry for the CRS in the presence and absence of the Arg guanidino group occupying position 2 of Ang II. These findings suggest that Tyr is preferred over His for bearing the negative charge and that the CRS is stabilized by the guanidino group. Recent crystallography studies provided details of the binding of nonpeptide angiotensin receptor blockers (ARBs) to the Ang II type 1 (AT1) receptor, and these insights were applied to Ang II. A model of binding and receptor activation that explains the surmountable and insurmountable effects of Ang II analogues sarmesin and sarilesin, respectively, was developed and enabled the discovery of a new generation of ARBs called bisartans. Finally, we determined the ability of the bisartan BV6(TFA) to act as a potential ARB, demonstrating similar effects to candesartan, by reducing vasoconstriction of rabbit iliac arteries in response to cumulative doses of Ang II. Recent clinical studies have shown that Ang II receptor blockers have protective effects in hypertensive patients infected with SARS-CoV-2. Therefore, the usage of ARBS to block the AT1 receptor preventing the binding of toxic angiotensin implicated in the storm of cytokines in SARS-CoV-2 is a target treatment and opens new avenues for disease therapy.

## 1. Introduction

**Figure 1:**
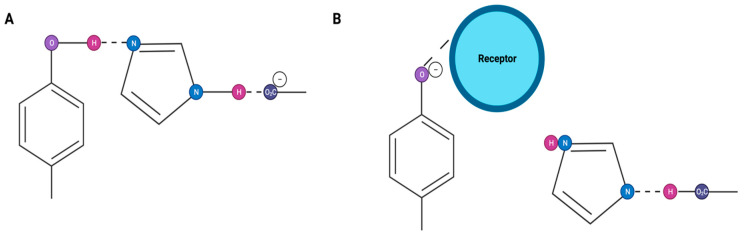
Angiotensin CRS showing the movement of charge from carboxylate to tyrosinate (**A**); interaction of tyrosinate with receptor (**B**).

The octapeptide angiotensin II (Ang II) (DRVYIHPF) acts on the Ang II type 1 (AT1) receptors in a variety of vascular smooth muscle tissues, eliciting a contractile response. This results in an increase in blood pressure. Several lines of evidence suggest that the interaction of Ang II with its receptors involves a charge relay mechanism (CRS) [1]. Accordingly, folding of the peptide in the hydrophobic membrane receptor environment brings together the Tyr4, His6, and Phe8 side chains of the peptide in a concerted interaction. This results in the transfer of the negative charge at the C-terminal carboxylate to the Tyr4 hydroxyl group via the His6 imidazole (Figure 1), which is analogous to serine proteases. The resulting generated tyrosinate species, which can be chemically and spectroscopically detected [2], are thought to have a pivotal role not only in activating the receptor but also in the mechanism of receptor desensitization. Thus (Sar1 Tyr(Me)4)Ang II (sarmesin) is a surmountable competitive antagonist, illustrating the role of the Tyr hydroxyl for agonist activity/receptor activation. In contrast (Sar1 Ile8)Ang II (sarilesin) is an insurmountable blocker, like angiotensin receptor blockers (ARBs), which becomes surmountable after the methylation of its Tyr hydroxyl group [2,3]. The above-mentioned angiotensin peptides are classified as a superagonist (Sar1AngII), surmountable antagonist(Sarmesin), and insurmountable inverse agonist (Sarilesin).

In the present report, we applied molecular modeling calculations to gain further insight into details of the CRS. In particular, the architecture of the triad of interacting groups is such that more than one mechanism for generating the tyrosinate species could exist. Furthermore, the Arg^2^ guanidino group of Ang II appears to have a central role in chaperoning the CRS [3,4,5,6]. At the receptor, the role of the Arg2 guanidino group (which chaperones the CRS in ANG II) appears to be substituted by R167 of the receptor (whereupon Arg2 of ANG II presumably interacts with a negative charged group(s) on the receptor). Likewise, the Tyr4 hydroxyl of the CRS in ANG II may also exchange with the Y35 hydroxyl of the receptor, thereby eliciting the response mechanism—as elaborated upon in Figure 6. 

## 2. Materials and Methods

### 2.1. Materials

Candesartan (Cat#SML0245) was purchased from Sigma Aldrich (St Louis MO, USA), and human Ang II (Cat#51480) was purchased from Mimotopes (Melbourne, VIC, Australia).

### 2.2. In Silico Molecular Experiments

Molecular mechanics was used for molecular dynamics simulations with heating and cooling phases to obtain a low energy starting conformation. Thereafter, semiempirical AM1 energy calculations were conducted to refine energy minima values. Calculations were carried out on isolated side chains from Ang II, as well as the whole molecule. The uncharged and charged forms of the amino acid side chains of Tyr and His were represented by phenol/phenolate and imidazole/imidazolate, and the C-terminal carboxylate was represented by acetic acid/acetate. At a physiological pH, the amino acid side chains of Tyr and His are normally uncharged. In contrast, the carboxylate group is negatively charged, and the Arg guanidinium group carries a positive charge. For the purposes of the present calculations, the carboxylate was considered to be a weak acid (pKa = 3–4) that is able to be protonated by a local donor. However, the guanidinium group is considered to be too strongly basic (pKa = 12–13) to surrender a proton.

### 2.3. In Vitro Animal Experiments

#### 2.3.1. Animal Model and Ethics Approval

Male New Zealand White rabbits (*n* = 4) at 7 weeks of age were purchased from Flinders City University (SA, Australia). The animals were individually housed at the Victoria University Werribee Campus Animal Facilities until 16 weeks of age. Upon arrival, animals were given a 7-day acclimatization period. Animals were kept on a 12-h day/night circadian rhythm cycle, and they were maintained at a constant temperature of 21 °C and relative humidity level between 40 and 70%. Food and water were supplied ad libitum. All experimental procedures were conducted in accordance with the National Health and Medical Research Council ‘Australia Code of Practice for the Care and Use of Animals for Scientific Purposes’ (8th edition, 2013), and they were approved by the Victoria University Animals Ethics Committee (VUAEC#17/013).

#### 2.3.2. Sedation and Anesthesia Protocol

Prior to the administration of inhalant anesthesia, animals were sedated using a 0.25 mg/kg subcutaneous injection of medetomidine at the ‘scruff’ or base of the neck. Once sedated, animals were transferred into an induction chamber and anaesthetized using 4% isoflurane. Once anesthetized, an incision was made at the lower abdomen and the subcutaneous tissue and lower abdominal muscles were dissected to expose the inferior vena cava. The inferior vena cava was perforated, and exsanguination was allowed for 3 min or until loss of color and dilation of pupils was observed. A T-tube was introduced distal to the aortic arch and flushed with a cold, oxygenated Krebs–Henseleit solution (Krebs). Both iliac arteries were retrieved from each animal, and, under a light microscope, they were cleaned of fat and connective tissue and dissected into 2–3 mm rings in preparation for isometric tension analysis.

#### 2.3.3. Drug Incubations and Isometric Tension Analysis

Rings were immediately sequentially transferred into adjacent organ baths (Zultek Engineering, VIC, Australia) filled with 5 mL of Krebs, marinated at 37 °C, and continuously bubbled with 95% carbogen. Rings were allowed to acclimatize for 15 min, and they were then mounted between two metal organ hooks attached to force displacement transducers, stretched to 0.5 g, and allowed to equilibrate for 15 min. Rings were re-stretched, refreshed, and equilibrated for a further 15 min. At this time, iliac artery rings were (a) left to rest for 10 min (control; *n* = 4), (b) incubated with candesartan (10^−5^ M) for 10 min to serve as an internal control (candesartan; *n* = 3), or (c) incubated with a novel biasartan (10^−5^ M) for 10 min (BV6(TFA); *n* = 3). To determine the ability of the newly formulated bisartan to behave as an ARB, an AngII dose response (from 10^−12^ to 10^−5^ M) was performed. To determine standardized vasoconstriction abilities, rings were washed, allowed to return to baseline, and constricted with KPSS (125 mM).

#### 2.3.4. Statistical Analysis

GraphPad prism (Version 8.4.2, GraphPad Software Incorporated, San Diego, CA, USA) was utilized to analyze isometric tension data. The significant *p*-value was set at *p* < 0.05, and a two-way ANOVA followed by Sidak’s multiple comparisons post hoc test was performed to determine significance on isometric tension analysis data. All data are represented as mean ± SEM.

## 3. Results and Discussion

Semiempirical energy calculations for the isolated triads were first carried out in the absence of Arg, and the calculated energies are given in Table 1. The sum of the heats of formation for the individual components of the triad was compared with the heats of formation for the complex of interacting triads. The difference in the heats of formation between non-interacting and interacting triads represented the net stabilization energy for complex formation. The computed interaction energies in Table 1 illustrate that the acetate group preferred to bear the negative charge, the phenol and imidazole groups were similarly less inclined, and the energy barrier to charge transfer among the three groups was relatively low (~5 kcal/mole). This suggests that the appearance of another influence such as a receptor-based group in the vicinity could readily influence the outcome of charge transfer and determine the resulting location of the negative charge. In accordance with this general concept, fluorescence lifetime studies on Ang II in receptor-simulating environments have demonstrated the presence of tyrosinate anions that become increasingly stabilized as the dielectric constant of the environment decreases [2].

### 3.1. The Stabilizing Role of Arg in Angiotensin II Conformation

**Figure 2:**
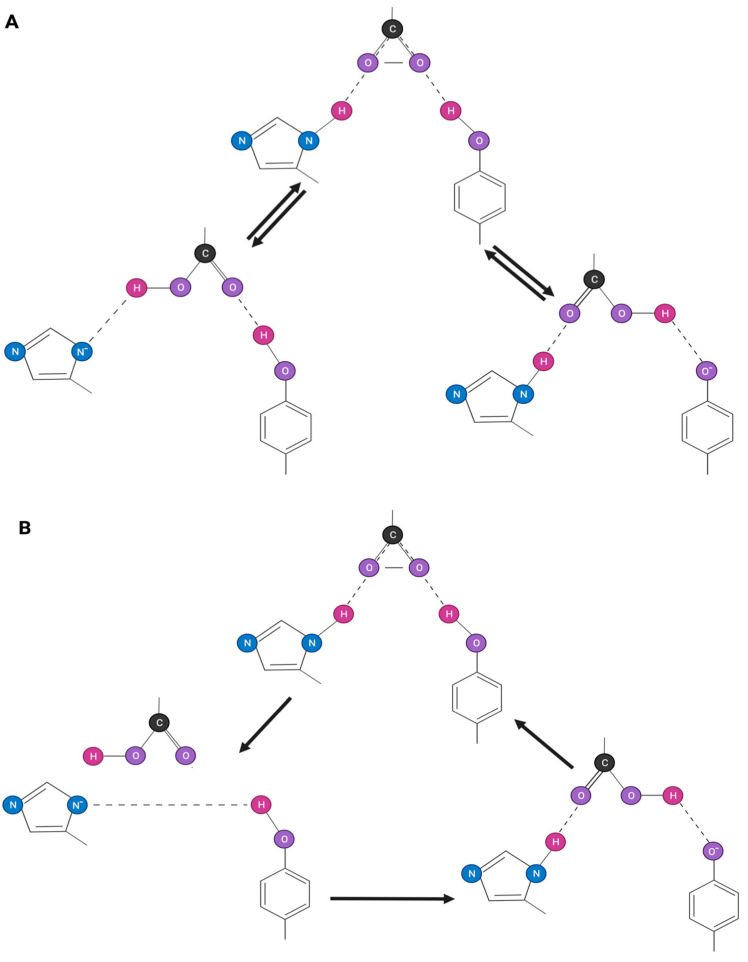
Angiotensin II charge relay system showing the movement of charge from carboxylate to tyrosinate. (**A**) “Swing” mechanism in which the C-terminal carboxylate group on angiotensin abstracts a proton directly from either the imidazole of His^6^ or the hydroxyl of Tyr^4^. (**B**) “Roundabout” or charge relay mechanism in which the C-terminal carboxylate group of angiotensin abstracts proton from the imidazole of His^6^, which, in turn, abstracts the hydroxyl proton of Tyr^4^.

Nuclear Overhauser effect (NOE) connectivities from NMR studies have suggested that the N-terminal part of Ang II is located near the proposed CRS [3]. Since the proximity of the Arg2 guanidino to the CRS could influence the outcome of charge transfer within the triad of interacting groups, it was of interest to calculate the energetics of the quaternary complex comprised of the triad plus guanidino group. Accordingly, the four individual groups were placed in proximity and allowed to optimize until an energy minimum was reached (Table 2). As expected, the introduction of the positively charged guanidino group to the negatively charged triad increased the stabilization energy of the overall complex (Table 2) compared to the triad alone (Table 1). In addition, the guanidino group disrupted the geometry of the charge relay triad, as shown schematically in Figure 1, through its insertion (together with the carboxylate) between the phenol and imidazole groups (Figure 2). The energy barrier for phenolate formation increased from ~5 kcal/mol for the triad (Table 1) to ~15 kcal/mol for the quaternary complex (Table 2), making charge relay more difficult in the presence of the guanidino group. However, the geometry of the functional groups was such (Figure 2) that it appears possible to generate phenolate anions through the direct interaction of carboxylate with phenol without invoking the imidazole group as an intermediate. On the other hand, the energy calculations shown in Table 2 illustrate that the carboxylate would prefer to abstract the imidazole proton (−66.2 kcal/mol) rather than the phenol proton (−61.1 kcal/mol), leaving open the possibility for a charge relay mechanism as originally proposed (Figure 1), though with the Arg2 guanidino group acting as chaperone.

### 3.2. Backbone and Mobility of Side Chains in Angiotensin II

These calculations (Table 1 and Table 2) explained the unlimited mobility of the functional groups and may not be representative of the situation for Ang II where the side chains are tethered to the peptide backbone and may not be able to access such conformational space. However, NMR studies on the superagonist (Sar^1^)Ang II in receptor-simulating environments [3] have shown the proximity of the three aromatic rings together with the N-terminus, and when the NOE constraints obtained from these NMR studies were included in the modeling process, the conformation shown in Figure 2 emerged. In this conformation, there was electrostatic interaction of the functional groups in a parallel manner to that found for the untethered groups (Figure 2). Surprisingly, these findings de-emphasize the role played by the peptide backbone in creating steric constraints and show that the backbone does not affect the mobility of the sidechains or prevent the formation of the optimal geometric arrangement of functional groups. In fact, energy calculations carried out on the intact (Sar)Ang II molecule indicated that the difference in the heats of formation for the carboxylate (−199 kcal/mol) and tyrosinate (−206 kcal/mol) forms of the peptide was only 7 kcal/mol, suggesting that the energy barrier to charge transfer for the whole molecule was less than for the untethered side chains. This would seem to indicate that there may be another contributing factor in the intact peptide that facilitates charge transfer—possibly the N-terminal amino group.

### 3.3. Angiotensin II Receptor Blockers

**Figure 3:**
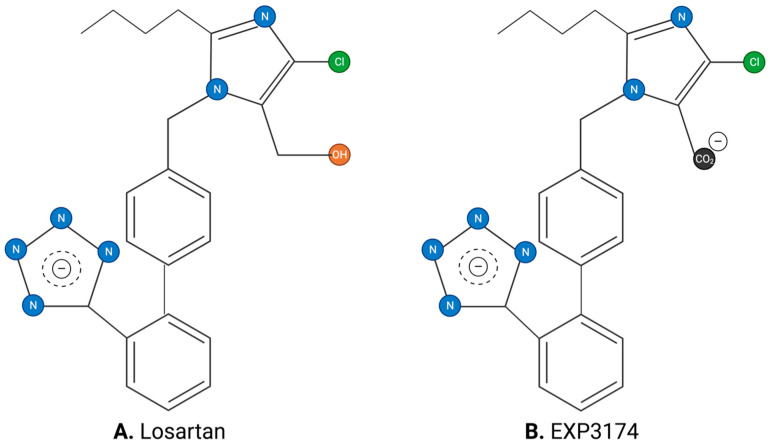
Angiotensin receptor blockers: **(A)** losartan (surmountable) and **(B)** EXP3174 (insurmountable).

The ARBs have provided important drugs for treating cardiovascular diseases, such as hypertension. The first nonpeptide ARB reported was the surmountable antagonist losartan, which is metabolized in vivo to the insurmountable inverse agonist EXP3174 (Figure 3). Most therapeutically useful ARBs contain an imidazole-based carboxylate group like EXP3174 (e.g., valsartan, olmesartan, and candesartan), which imparts inverse agonist effects (biased agonism). Inverse agonism occurs when the nature of the ligand (as well as how it interacts with the receptor) prevents the receptor from binding the G protein and dimerizing (resulting in smooth muscle contraction), instead causing the binding of an alternative second messenger (resulting in relaxation).

### 3.4. Crystallography of Angiotensin Receptor Blockers/Angiotensin II Type 1 Receptors Complex

**Figure 4:**
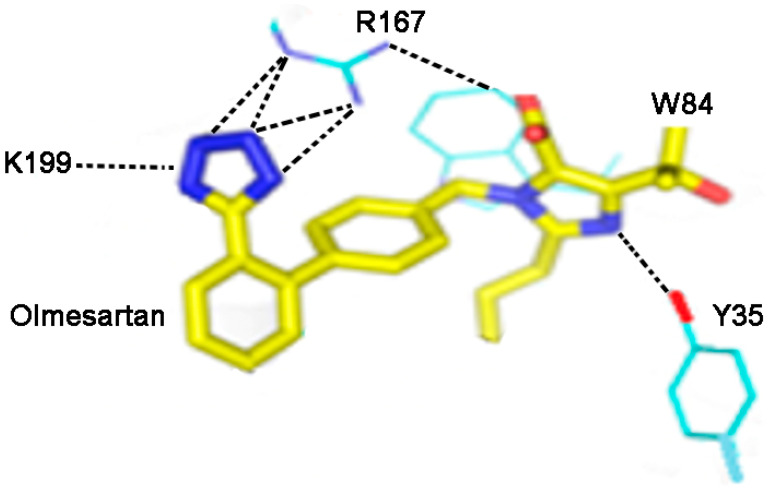
Interactions between receptor and olmesartan were determined from crystal structure [7]. The critical interactions of olmesartan are with R167, W84, Y35 and K199 residues of AT1R (reproduced from, Zhang et al, *JBC,* 290, 29127-29139, 2015).

Crystallographic studies of ARBs bound to the AT1 receptor [7,8] have revealed some critical interactions between receptor and drug molecule. In particular, it has been found that the two anions present in all insurmountable ARBs, namely the imidazole carboxylate and the biphenyl tetrazole (Figure 3 and Figure 4), form salt bridges with the cationic guanidino sidechain of R167 of the receptor. In addition, the Y35 hydroxyl group of the receptor H-bonds to the imidazole N of the ARB [7]. These interactions (Figure 4) have revealed a unique network of charge interactions between ARBs and receptors that are characteristically similar to the CRS elaborated for Ang II (except that the carboxylate in ARB is tethered to the imidazole ring, creating an inductive effect on the imidazole N that accepts the phenolic proton of Y35 rather than a relay of charge per se). The similarity is so striking that it is tempting to speculate that tyrosinate is generated in Ang II at the receptor, not by direct interaction with the C-terminal carboxylate but via relay through the His^6^ imidazole. 

### 3.5. Effects of Tyrosine Methylation in Activity and Conformation

**Figure 5:**
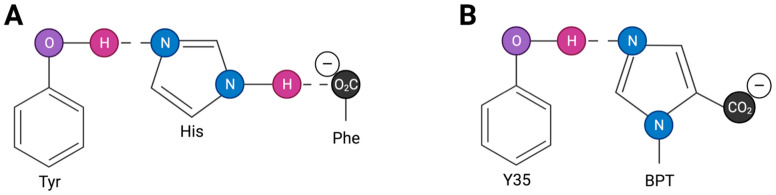
(**A**) Intra CRS in Ang II; (**B**) inter CRS in the complex AT1R/ARB olmesartan.

As outlined above, the guanidino group of Arg^2^ in Ang II appears to be important for chaperoning and maintaining the CRS, and this same interaction may be mimicked (replaced) by R167 of the receptor upon binding. A similar interaction was reproduced here for ARBs in the form of two salt bridges (carboxylate and tetrazole) with the R167 of the receptor (Figure 5A) (the tetrazole of ARB and the carboxylate of Ang II may also bind to K199 (Figure 4)). When ARB and Ang II structures are overlayed, the tyrosinate of Ang II corresponds to the carboxylate of ARB and the carboxylate of Ang II corresponds to the tetrazolate of ARB [1]. This orientation has been confirmed by structure–activity studies, which have revealed that removal of the negative charge by methylation of TyrOH in sarilesin has the same effect as the neutralization of the carboxylate in ARB (Figure 3) (i.e., changing both molecules from insurmountable into surmountable antagonists). Apparently the existence of a salt bridge with R167, which increases the strength of binding of ARBs to the receptor, is what differentiates an insurmountable antagonist from a surmountable one. Sarilesin, which is an insurmountable analogue that demonstrates negative cooperativity/inverse agonism identical to ARBs in many tissues [1], presumably affords the same salt bridge interaction with R167 as a direct consequence of the tyrosinate anion provided by the CRS. Accordingly, when the TyrOH of sarilesin is methylated, this salt bridge is converted to a weaker ion dipole bond, and the result is a surmountable antagonist [1].

### 3.6. Critical Interaction of AT1R 35Y with Angiotensin II and Angiotensin Receptor Blockers

**Figure 6:**
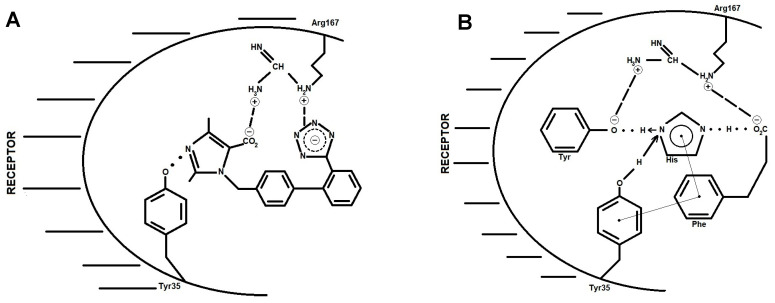
Binding of (**A**) ARB and (**B**) AngII to angiotensin AR1 receptor. (**A**) ARBs characteristically contain a carboxylate and a tetrazole group that form two salt bridges with Arg167 of the receptor, resulting in insurmountable blockade of Ang II. When the carboxylate anion is neutralized, as in losartan (CH2OH) or olmesartan (CONH2 in R239470), and changes the salt bridge to a weaker ion dipole bond, the molecule becomes a surmountable antagonist. (**B**) The charge transfer and separation created by the CRS allows Ang II to bind in a similar manner to ARBs (Figure 5A), though with tyrosinate replacing the carboxylate in ARBs and the C-terminal carboxylate standing in for the tetrazole of ARBs. Like ARBs, the peptide analogue sarilesin can form a salt bridge via its tyrosinate with R167 and is consequently an insurmountable blocker. In parallel with ARBs, the methylation of the TyrOH of sarilesin eradicates this salt bridge and converts it into a surmountable antagonist. In contrast, the presence of the Phe^8^ ring in Ang II provides agonist activity by attracting the receptor Y35 ring towards the CRS, eventually allowing the Y35 OH group to H-bond with the His^6^ imidazole N of Ang II (exactly equivalent to ARB binding in Figure 5A) and displacing the TyrOH of Ang II so that it no longer carries a charge and cannot form a salt bridge with the R167 of the receptor. This exchange is reversible and requires a cooperative interaction involving the binding of the G protein intracellular messenger and receptor dimerization. When there is no more available G protein (at supramaximal doses), Ang II can bind just like sarilesin and become an insurmountable blocker, invoking tachyphylaxis.

Interestingly, the methylation of the Tyr hydroxyl in Ang II results in a competitive surmountable antagonist (sarmesin), implying that tyrosinate is also required for agonist activity (in addition to its role for insurmountable blockade by sarilesin outlined above). Again, there is a repeating pattern when connecting receptor-binding interactions with bioactivity. What makes Ang II itself different from sarilesin is the Phe ring at the C-terminus—a structural difference that endows agonist activity. One possible explanation for this may be related to the critically important Y35, which is known to be essential for the binding of ARBs and Ang II [7]. In ARBs, the Y35 phenolic group bonds to imidazole N (Figure 5A and Figure 6A), and it follows that Y35 should also be in the right place to potentially interact with the imidazole N of His in Ang II. For sarilesin, Y35 may be unable to access the imidazole of His (without the assistance of other receptor-based groups) because of the complexity of the CRS interactions. However, in Ang II itself (Figure 6B), the presence of the Phe^8^ ring offers the possibility of a ring:ring interaction with Y35, which, in turn, could draw the Y35 ring closer to the CRS (probably reinforced by the preexisting Phe:His ring interaction in Ang II [3]). Note that an aromatic ring has a quadrupole moment, which allows it to form a slipped parallel plate or perpendicular plate electrostatic interaction with another ring; consequently, aromatic rings do not interact with hydrophobic sidechains, such as Ile^8^ in sarilesin, which is why they are not agonists. Indeed, it is entirely possible that the Tyr^4^ of Ang II can swap roles with Y35 of the receptor, and that this interchange is the basis for the agonist activation of the receptor (Figure 6B). Thus, the CRS may alternate from Ang II Tyr^4^ to receptor Y35 (on-off mass action), the latter option being reinforced by the concerted action of intracellular G-protein binding and receptor dimerization leading to the positive cooperativity (amplification) of the contractile response [9,10]. When the supply of G protein is exhausted (e.g., at supramaximal doses of Ang), this concerted mechanism for receptor activation can no longer occur, and Ang II may then bind like sarilesin and become an insurmountable blocker, thereby causing tachyphylaxis effects.

### 3.7. Surmountable and Insurmountable Blockers

In this model (Figure 5), the bioactivities of agonists, surmountable antagonists, and insurmountable blockers for both peptides and nonpeptides could be accounted for by an interaction with a single residue on the receptor. Thus, the quality of the bond between the ligand and the receptor R167 guanidino group determines the outcome, with (1) a strong salt bridge providing for insurmountable block/inverse agonism (sarilesin or ARB with carboxylate like EXP3174), (2) a weaker ion:dipole bond providing for surmountable antagonism (sarmesin, O-methyl-sarilesin, or ARB without carboxylate like losartan), and (3) disrupted (exchange) bonding (together with other cooperative factors) leading to agonist action (Ang II) [11]. 

### 3.8. Bisartans: A New Class of Sartans

**Figure 7:**
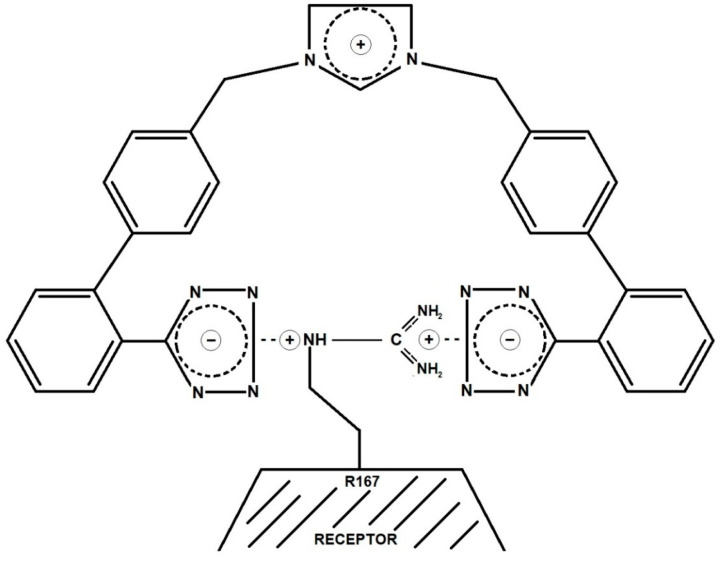
Binding of bisartan to AT1 receptor. The two tetrazolates react with the guanidino group of Arg167.

This model of receptor interaction (Figure 5) has enabled the development of more potent nonpeptide Ang II mimetics as potential drugs for treating hypertension and other cardiovascular diseases [12,13]. These new generations of drugs, called bisartans, contain two tetrazole groups (the carboxylate present in all insurmountable ARBs was replaced by its functional mimetic tetrazole) that are mounted on an imidazole template as biphenyl tetrazole groups. Accordingly, both tetrazole groups are available to form salt bridges with R167 on the receptor (as per Figure 5), creating an insurmountable blocker. Additionally, the imidazole cation is at the right distance to mimic the role of the Arg^2^ sidechain of Ang II and therefore provide an additional salt bridge to the receptor, which may explain the increased potency of bisartans (Figure 7). 

### 3.9. The Novel Bisartan BV6(TFA) Potently Blunts Angiotensin II-Mediated Vasoconstriction in Rabbit Iliac and Arteries

**Figure 8:**
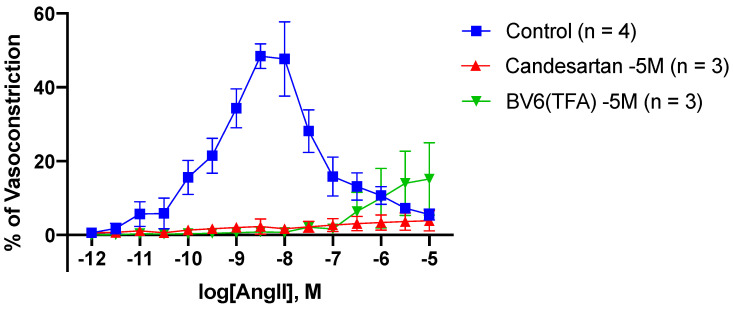
Inhibitory effect of BV6(TFA) and candesartan on angiotensin II-mediated vasoconstriction responses in rabbit iliac arteries. To determine the ability of BV6(TFA) to behave as an ARB, like candesartan, rabbit iliac arteries were incubated and then constricted using cumulative doses of Ang II. Candesartan and the novel bisartan BV6(TFA) were able to potently inhibit vasoconstriction responses to Ang II at doses [10^−9.5^ M] to [10^−7.5^ M] (mean ± SEM is shown; significance is presented in Table 3).

To evaluate the newly synthesized bisartan as an ARB mimetic, iliac artery rings collected from rabbits were incubated with BV6(TFA). An Ang II dose–response assessment was performed to determine the ability of BV6(TFA) to inhibit Ang II-mediated vasoconstriction (Figure 8). Vasoconstriction responses were then compared to control rings (no incubation) and internal control rings incubated with candesartan [14]. As expected, candesartan was able to potently inhibit vasoconstriction in response to cumulative doses of Ang II: from Ang II [10^−9.5^ M] (control: 21.49 ± 4.75% vs. candesartan: 1.72 ± 1.14%, ** *p* < 0.01) to Ang II [10^−7.5^ M] (control: 15.82 ± 5.25% vs. candesartan: 2.69 ± 1.73%, * *p* < 0.05) (Table 3). Interestingly, similar results were observed in rings incubated with BV6(TFA), as vasoconstriction was significantly inhibited to cumulative doses to Ang II when compared to control rings: from Ang II [10^−9.5^ M] (BV6(TFA): 0.49 ± 0.73% vs. control * *p* < 0.05) to Ang II [10^−7.5^ M] (BV6(TFA): 2.08 ± 1.12% vs. control: 28.10 ± 5.78%, *** *p* < 0.001) (Table 3). However, vasoconstriction was seen at Ang II [10^−6.0^ M] to Ang II [10^−5.0^ M], but no significance was determined. Furthermore, no significance was observed between candesartan, a known AT1 receptor antagonist [14], and BV6(TFA). This suggests that BV6(TFA) may act on the AT1 receptor, potentially eliciting anti-hypertensive abilities as a treatment for cardiovascular diseases. However, further studies are required to determine if the vasoconstriction shown at the higher doses of Ang II could be reduced or blocked by increasing/decreasing the dose of BV6(TFA).

### 3.10. Relevance to COVID-19

ARBS and angiotensin I-converting enzyme inhibitors (ACEi) were recently reported to protect hypertensive patients infected with SARS-CoV-2. Angiotensin-converting enzyme 2 (ACE2) and the renin–angiotensin system (RAS) inhibitors reduce excess AngII and increase the antagonist heptapeptides alamandine and aspamandine, which counterbalance Ang II and maintain homeostasis and vasodilation [13]. In particular, the CRS of Ang II described in the study well-explains tyrosine-based ligand–receptor interactions and can be applied to the new aggressive SARS-CoV-2 mutations, which is a pressing issue. Tyrosine seems to be a major player in this issue, and the N501Y mutation of the UK variant B1.1.7 is an example that shows that tyrosine is a much better binder with ACE2 than asparagine. The RAS and in particular ACE2 are the entry points of the virus, and this study significantly contributes to the understanding of the molecular mechanisms of Ang II and, subsequently, the driving forces that lead to the infectivity and transmissibility of the new mutations. We already reported the first evidence for the benefit of ARBs as promising repurposed drugs to treat infection in recent publications [13,15,16,17,18,19].

The protective effects of ARBs against SARS-CoV-2 infection was further validated and confirmed in a recent open multicenter randomized clinical trial using the ARB telmisartan and has been postulated to treat coronavirus 2019 (COVID19)-induced lung inflammation [20]. Telmisartan is the strongest binder among all ARBs, and it appears to disrupt the binding between the receptor-binding domain of the spike protein and ACE2 [21]. The mutations in SARS-CoV-2 have led to stronger binding between the receptor-binding domain of the spike protein and ACE2, resulting in increased infectivity [22]. Telmisartan which is large and rich in pi electrons may disrupt this binding, leading to protection from infection. Overall, the elevation of Ang II in the RAS seems to play a pivotal role in promoting inflammation and tissue injury. The hypothesis of the involvement of the RAS in the inflammatory process triggered by the entry of SARS-CoV-2 into tissues (primary site being the lungs) considers that the downregulation of ACE2 causes an imbalance in the RAS that results in an elevation of Ang II concentrations (pro-inflammatory) and the cytokine storm in COVID19 patients. ARBs upregulating ACE2 and decreasing Ang II may comprise an answer to COVID19.

## 4. Conclusions

The present study supports the occurrence of a charge transfer system in angiotensin and elaborates on the geometry of the interaction of the functional groups. The introduction of the Arg side chain into the network alters the geometry of the charge relay interaction and has a stabilizing influence on the folded compact charge transfer conformation. If this conformation approximates that which is present when Ang II binds to its receptor, then the Arg guanidino group can be visualized to act as a chaperone for the angiotensin CRS. Mutation-bioactivity studies on AT1 receptors and crystallographic data for ARB binding to the AT1 receptor have implicated R167 (necessary for insurmountable effects) and Y35 (essential for binding of Ang II and ARB) as anchor residues on the receptor [7]. By forming a salt bridge with R167, the insurmountable Ang II analogue sarilesin, as well as the insurmountable nonpeptide ARBs, apparently lock the receptor into a conformation that cannot bind G protein but can bind an alternate messenger and lead to inverse agonism [7,8]. For the receptor binding of Ang II itself, we propose a model in which the Arg2 of Ang II, which chaperones the CRS, can be replaced by the R167 of the receptor upon binding. This interaction sets up a situation where the Tyr4 of the CRS can be replaced by the Y35 of the receptor, creating an intermolecular exchange mechanism for activating the receptor response mechanism. The mutation Y35–A35 disrupts and abolishes the binding [7]. The Phe8 ring of Ang II, which is essential for agonist activity, may have a functional role in guiding the Y35 ring through quadrupolar ring:ring interactions into the correct alignment for receptor activation. Such considerations have led to the development of a new generation of nonpeptide Ang II mimetics as potential drugs for treating hypertension and other cardiovascular diseases, including bisartans [12,13,19]. Recent clinical findings from hospitalized hypertensive patients infected by SARS-CoV-2 have shown a protective effect against the infection by the virus and reduction of morbidity and mortality. The crystal structure of the RBD spike protein/ACE2 complex revealed critical interactions that link the two chains. This binding is strengthened by mutations that stabilize the complex. The disruption of the binding is a key to treatment therapeutics. Thus far, researchers have reported a number of repurposable drugs that interfere in the interface, disrupting binding and consequently decreasing infectivity and transmissibility. One of them is telmisartan, as postulated in recent clinical trial. ARBs generally seem to be tentative repurposed therapeutics for SARS-CoV-2 infection, as shown by clinical and in silico studies. Further studies are required to confirm these early findings. 

## Figures and Tables

**Table 1:** Calculated heats of formation (kcal/mol) for triad complexes.

Complex	Sum of Components	Computed Interaction	Stabilization
Phenol–Imidazole–Acetate	−98.4	−129.3	30.9
Phenol–Imidazolate–Acetic acid	−110.0	−124.2	14.2
Phenolate–Imidazole–Acetic acid	−104.3	−125.4	21.1

**Table 2:** Calculated heats of formation (kcal/mol) for quaternary complexes.

Complex	Sum of Components	Computed Interaction	Stabilization
Phenol–Imidazole–Acetate	+53.3	−76.2	129.7
Phenol–Imidazolate–Acetic acid	+41.9	−66.2	108.1
Phenolate–Imidazole–Acetic acid	+47.6	−61.1	108.7

**Table 3:** Significance of vasoconstriction in response to cumulative doses of angiotensin II between control, candesartan, and Bv6(TFA) incubations obtained from Figure 8.

log[AngII, M]	Control vs. BV6(TFA)	Control vs. Candesartan	BV6(TFA) vs. Candesartan
−12.0	No significance	No significance	No significance
−11.5	No significance	No significance	No significance
−11.0	No significance	No significance	No significance
−10.5	No significance	No significance	No significance
−10.0	No significance	No significance	No significance
−9.5	*, *p* < 0.05	**, *p* < 0.01	No significance
−9.0	****, *p* < 0.0001	****, *p* < 0.0001	No significance
−8.5	****, *p* < 0.0001	****, *p* < 0.0001	No significance
−8.0	****, *p* < 0.0001	****, *p* < 0.0001	No significance
−7.5	***, *p* < 0.001	****, *p* < 0.0001	No significance
−7.0	No significance	*, *p* < 0.05	No significance
−6.5	No significance	No significance	No significance
−6.0	No significance	No significance	No significance
−5.5	No significance	No significance	No significance
−5.0	No significance	No significance	No significance

## Data Availability

The data presented in this study are available on request from the corresponding author.
